# Ecological Effects of Benzyl Chloride on Different Korean Aquatic Indigenous Species Using an Artificial Stream Mesocosm Simulating a Chemical Spill

**DOI:** 10.3390/toxics9120347

**Published:** 2021-12-09

**Authors:** Soo-Yeon Kim, Seong-Hwan Park, Dae-Wook Kim, Won Noh, Sang-Jun Lee, Hee-Jin Jeong, Jong-Bin Park, Yeong-Ji Gwak, Jin-Woo Park, Dong-Hyuk Yeom

**Affiliations:** 1 Gyeongnam Branch Institute, Korea Institute of Toxicology (KIT), Jinju-si 52834, Korea; sykim@kitox.re.kr (S.-Y.K.); seonghwan.park@kitox.re.kr (S.-H.P.); dwkim@kitox.re.kr (D.-W.K.); won.noh@kitox.re.kr (W.N.); sjlee2013@kitox.re.kr (S.-J.L.); heejin.jeong@kitox.re.kr (H.-J.J.); jongbin.park@kitox.re.kr (J.-B.P.); yeongji.gwak@kitox.re.kr (Y.-J.G.); parkjw@kitox.re.kr (J.-W.P.); 2 Department of Energy Environmental System Engineering, Graduate School, University of Seoul, Seoul 02504, Korea

**Keywords:** Korean indigenous species, risk assessment, benzyl chloride, artificial stream

## Abstract

In this study, an artificial stream mesocosm consisting of a head tank, faster-flowing riffle section, gravel section, pool section, lower-run section, and tail tank was installed to simulate a chemical spill in a river. The responses of freshwater periphyton algae, crustacea (*Moina macrocopa*), freshwater worm (*Limnodrilus hoffmeisteri*), benthic midge (*Glyptotendipes tokunagai*), and fish (*Zacco platypus* and *Aphyocypris chinensis*) were observed after exposure to benzyl chloride (classified as an accident preparedness substance, APS) at concentrations of 1, 2, and 4 µL/L for 22.5 h. Higher concentrations increased the inhibition (photosynthetic efficiency decrease) of periphyton algae and the mortality of *M. macrocopa*, whereas the reproduction of the female cladoceran decreased in the 4 µL/L treatment. Mortality of fish did not occur or was lower (≤20%) at all concentrations; however, toxic symptoms were observed for some time after chemical exposure termination and later, symptoms receded. *G. tokunagai* mortality increased at all concentrations except the control after seven days, and no significant toxic effects were observed in *L. hoffmeisteri*. The hazardous concentration of benzyl chloride was calculated as 94 µg/L. This study showed the different sensitivities of each species to benzyl chloride. The findings can assist in environmental risk assessment of APSs after chemical spills to protect Korean aquatic species.

## 1. Introduction

Benzyl chloride is used in various industrial purpose such as a chemical intermediate in the manufacture of certain dyes and pharmaceutical products, perfume, flavor products and as a photographic developer. It can be used in the manufacture of synthetic tannins and as a gum inhibitor in petrol [1] and the world consumption of benzyl chloride has increased steadily due to those diverse applications [2]. Industrial chemicals have the potential to cause chemical accidents, and the frequency of chemical accidents has increased with the increasing use of such chemicals [3]. According to the FACTS (Failure and Accidents Technical Information System) website, more than 25,700 industrial accidents occurred globally in the past 90 years, and there were five accidents involving benzyl chloride [4]. Recently, industrial chemical spills have continued to occur in Korea [5], and a total of 661 cases have occurred since 2014 [6]. A large chemical accident of hydrogen fluoride leak occurred in 2012, which has raised concerns regarding chemical safety, re-emphasizing hazardous chemical management [7]. Consequently, the Toxic Chemical Control Act (TCCA) of Korea was revised in 2015, and 97 substances were classified as accident preparedness substances (APSs). APSs are highly likely to be involved in chemical accidents because of their strong acute toxicity or explosiveness, and they were included in the Korean TCCA to protect aquatic ecosystems from chemical accidents [8].

Benzyl chloride is one of the concerning APSs by Korean authorities and there are several potential ways in which it could be involved in a chemical accident. Hong et al. proposed a chronic health risk assessment method for multi-media exposure of benzyl chloride by chemical accident scenario [9]. Depending on the circumstances of an accident, benzyl chloride can be released into aquatic ecosystems, and hence, it is necessary to assess the toxicological effects on aquatic organisms. Generally, a chemical spill releases large amounts of chemical into river over a short period, which may cause potential adverse effects, such as delayed (chronic) toxicity, as well as immediate (acute) toxicity to aquatic organisms. Therefore, evaluating the ecological risks arising from these chemical accidents is necessary [10].

Laboratory toxicity tests are fundamental and common tools to evaluate ecotoxicological effects due to contaminants; however, they are insufficient to reflect the real river conditions. Thus, interpreting the results of monitoring studies, particularly in rivers, is often complicated. Artificial stream mesocosms provide an opportunity to advance understandings of ecological phenomena that occur in rivers as the environmental conditions can be more realistic than those in laboratory toxicity tests, but more controlled than in field studies [11]. Thus, mesocosm studies have been suggested for ecological risk assessments [12,13], and the fate and effect of hazardous chemicals in rivers can be evaluated using artificial stream mesocosms without causing any chemical damage to the real aquatic environment [14]. Such studies can assess the environmental impacts of potential stressors, and link laboratory and field conditions. Since the 1990s, various stream and pond mesocosm studies have been applied for assessing the risks posed by pesticides [15,16,17,18,19,20]. Besides pesticide studies, most mesocosm research related to chemical spills has focused on oil spills in marine ecosystems [21,22,23]. Moreover, recently, an increased number of mesocosm studies have been conducted to evaluate the impacts of microplastics on aquatic organisms [24,25].

Exposure to chemicals during a chemical spill in a river occurs instantaneously and the concentration of the chemicals, subsequently, decreases, primarily by dilution. However, replicating this scenario for research is difficult and thus, modeling approaches have been developed [26]. Acute toxicity data derived from standard laboratory exposure conditions with a short duration could provide valuable information that can be used for risk assessment; however, these conditions do not represent direct chemical spills occurring in the environment. Chapman et al. [27] reported that applying safety factors to acute toxicity data with continuous exposure does not provide toxicity values for short-term exposures (for several hours) and may overestimate the risks. Bejarano and Farr [28] developed an estimation program to overcome this problem; however, this program is based on existing toxicity data using international standard species, and autochthonous species data were not included. Additionally, the lack of toxicity data for some chemicals has limited the accuracy of evaluations during chemical spills.

Accordingly, in the present study, an artificial stream mesocosm system that included six Korean indigenous aquatic species (the fish: *Zacco platypus* and *Aphyocypris chinensis*; the crustacea: *Moina macrocopa*; periphytic alga; the freshwater worm: *Limnodrilus hoffmeisteri*; and the benthic midge: *Glyptotendipes tokunagai*) was installed to simulate the effects of benzyl chloride in real ecosystems. It was assumed that a river was exposed to benzyl chloride after a chemical accident. Subsequently, the responses of the above-mentioned species were observed. To the best of our knowledge, this is the first stream mesocosm study performed on benzyl chloride using indigenous Korean species to simulate chemical spills in Korea. Therefore, this study focused on evaluating the individual responses of each species when chemical spills occur and improving the understanding of different sensitivities among Korean species to chemicals. The present study intends to provide reliable and relevant ecotoxicological data, namely the no observed effect concentration (NOEC), for indigenous Korean species in real environments. Furthermore, hazard concentration (HC) was calculated using the NOEC for each species. This artificial stream mesocosm study can serve as a potential predictive tool for hazard/risk assessment of benzyl chloride.

## 2. Materials and Methods

### 2.1. Artificial Stream Mesocosm System

The artificial stream system consisted of five glass sections with a head tank, namely an upper riffle section, a lower run section, a pool section, a lower section, and a tail tank (Table 1). Each section was positioned to gradually decrease in height to reflect the characteristics of an actual stream. Dechlorinated tap water was passed through a membrane filter and a high-grade activated carbon filter, and introduced into the head tank at a rate of 5 L/min. To stabilize the artificial stream, the substrates were placed in each section and dechlorinated tap water was flowed through the system during a 30-day stabilization period. Test organisms (except periphyton algae) were introduced for acclimation during system stabilization. Details regarding each section of the artificial steam mesocosm are provided in Table 1.

### 2.2. Test Organisms

The Organization for Economic Cooperation and Development (OECD) guidelines [29] recommend that microcosm and mesocosm studies should focus on taxonomic groups concerned with lower-tier risk assessment. Accordingly, we selected test species belonging to five important taxonomic groups, namely periphytic algae, the cladoceran *M. macrocopa* (Arthropoda, Branchiopoda), the worm *L. hoffmeisteri* (Annelida, Oligochaeta), the midge *G. tokunagai* (Arthropoda, Chironomidae), and the fish *Z. platypus* and *A. chinensis* (Table 2).

Both fish species (*Z. platypus* and *A. chinensis*), *M. macrocopa* and *G. tokunagai* are indigenous to and widely distributed in Korea. Kim et al. [30] mentioned that *M. macrocopa* is an appropriate surrogate species for assessing toxic effects because of its abundance in the agriculture environments of Korea, and it also has economic importance, as reproduction assays can be conducted in a short period owing to its short life cycle. The average life span of *M. macrocopa* is approximately 10 d [30] and it has been observed that individuals continued to grow until 12 d, after which a steep decrease in their abundance was observed [31,32]. *M. macrocopa* is the most abundant cladoceran in Korea and the sensitivities of *M. macrocopa* and *D. magna* have been compared by several researchers [30,33]. Cho et al. [33] reported that *M. macrocopa* showed a higher sensitivity than *D. magna* for organic extracts of sediment from Korean streams. The mortality of *M. macrocopa* was 85% compared with the mortality of *D. magna* of 30%. Moreover, *Z. platypus* was identified as the most abundant species on the Korean Peninsula [34] and has been used as a target indicator to assess regional ecological health [35,36]. *G. tokunagai* is distributed widely in urban streams throughout East Asia and has been recommended for use as a water quality indicator in Korea [37].

In this study, the test species were introduced into each section for stabilization for 1–30 d before chemical exposure. Pebbles with periphytic algae were sampled from a natural river and then placed on top of the tile section to facilitate attachment of the algae onto the tiles for 30 d (three rows of 20 non-glazed tiles per tank), respectively. Acclimation for periphyton algae began at the same time as system stabilization and other species were introduced into the system in sequence. *Z. platypus* and *A. chinensis* (standard length: 3–4 cm) were obtained from the Institute of Biodiversity Research (Jeonju-si, Korea) and acclimated for two weeks prior to exposure in each section (pool and tail tank). Approximately, 240 2nd instar larvae of *G. tokunagai*, which were cultured in the laboratory, and approximately 240 *L. hoffmeisteri* adults were obtained from an aquatic animal supplier (Alpha Fish, Yeosu-si, Korea) Both species were introduced into the lower section with sand/sediment. *G. tokunagai* were acclimated for one day and *L. hoffmeisteri* were acclimated for seven days before staring of chemical exposure. *M. macrocopa* were cultured in the laboratory and five animals (age: <24 h) per replicate were introduced into a glass chamber with a mesh cover. Newly hatched *M. macrocopa* were introduced into the system without any acclimation period. After the acclimation period, all species were exposed to benzyl chloride during the same period of 22.5 h but then organisms were observed during different post-exposure periods. The information for each test species is summarized in Table 2.

### 2.3. Test Chemical and Exposure Scenario

Benzyl chloride used in this study was purchased from Sigma-Aldrich (CAS No.: 100-44-7, purity: 98.5%). Its solubility was 525 mg/L at 25 °C, log Kow was 2.30, and vapor pressure was 123 mmHg at 25 °C [38]. The exposure scenario, involved a simulated case of chemical leakage into the river upstream, from a chemical tank truck. An alternative scenario selection method was based on the Technical Guidelines on Accident Scenario Selection of the National Institute of Chemical Safety [39]. A worst-case scenario of acute toxic effects of 100% mortality of organisms was excluded. In the alternative scenario, the end-point concentration referred to the concentration that affects the environment after the chemical is released from the source. The endpoint concentration concept was used to calculate the certain concentration at the point at which the chemical that was discharged from the tank entered the water system. In addition, it was assumed that a certain concentration was maintained for a while in this study. Exposure concentrations were calculated using three acute toxicity (LC_50_) values from our preliminary acute toxicity test (unpublished data) in accordance with the endpoint concentration criteria of the alternative scenario. The highest concentration was determined based on this concept, and the lower concentrations were determined to give factor of 2. The exposure time to maintain the concentration was calculated using the amount and flow rate of the leaked chemicals it was 22.5 h in this study.

### 2.4. Artificial Stream Mesocosm Study

#### 2.4.1. Test Chemical Exposure

Four artificial stream mesocosms (one control and three treatments) established simultaneously. The exposure concentrations of benzyl chloride were 1, 2, and 4 µL/L, and the exposure time was 22.5 h based on the leaked chemical amount. Aliquots of 5 µL, 10 µL and 50 µL of benzyl chloride were released directly into water (5 L/min) using a syringe pump for each concentration. Benzyl chloride was detected after 1 h from the surface water of all systems and the measured concentrations were 15.5–48.6% of nominal concentrations after 5 h. After the 22.5 h of exposure duration, only clean dechlorinated tap water was introduced into the system during the 28-day post-exposure period.

#### 2.4.2. Observation of Test Organisms

A summary of test organisms is presented in Table 2.

- Periphyton algae

To identify the effect on the algae biomass and photosynthesis, chlorophyll-a concentration, dry cell weight (DCW) and quantum yield were measured in this study. Periphyton algae were collected from the tiles (surface area per tile: 75.69 cm^2^) in the riffle section on days −1, 0, 1, 7, 14, 21, and 28. Four tiles were randomly sampled from each mesocosm; subsequently, periphyton was scraped using a brush and mixed with distilled water to determine the chlorophyll-a concentration, dry cell weight and quantum yield. Chlorophyll-a concentration was measured using a standard method [40,41] and dry cell weight and quantum yield were measured according to the procedures used in a previous study [42].

- Crustacea

To investigate the survival and reproduction capacity of *M. macrocopa*, glass chambers were removed from each stream mesocosm daily. The chambers were returned to the mesocosm after the surviving parents were observed and the number of offspring was counted over 14 d.

- Fish

Fifty individuals of *Z. platypus* and *A. chinensis* were introduced in the pool and tail sections, respectively. Fish were maintained within each section because the depth did not allow the fish to swim to another section and also each section of the mesocosm system was designed to gradually decrease in height. Mortality and abnormalities (e.g., abnormal swimming behavior, and loss of equilibrium) of fish for each stream mesocosm were recorded daily. For determination of growth during the test period, the total length (using digital Vernier calipers) and wet weight of all surviving fish were measured on day 30 to compare the condition factor (K). Fulton’s condition factor was calculated using the following formula: K = 100 × W (wet weight, g)/L^3^ (total length, mm) [43].

- Chironomidae and freshwater red worm

Four subsamples (9 cm in diameter and 4 cm in depth) were collected from the lower sediment section to observe their effects on *G. tokunagai* and *L. hoffmeisteri*. The number of surviving individuals was counted in each subsample on day 0 (before chemical exposure) and on days 1, 7, 14, 21, and 28. Dry weights of *L. hoffmeisteri* in each subsample were measured after drying for 24 h at 60 ºC.

#### 2.4.3. Water Parameters and Chemical Analysis

Water temperature, dissolved oxygen (DO), pH, and conductivity of each mesocosm system were measured on day 0 (before chemical exposure) and on days 1, 7, 14, 21, 28, and 30. The temperature, pH, DO, and conductivity values were 22.8–27.9 °C (Table S1), 7.20–8.98, 7.82–9.92 mg/L, and 72–86 μS/cm, respectively (Instrument: Orion Star A211, Thermo Scientific). The water temperature was measured in the morning (9:00–10:00) or in the afternoon (15:00–17:00). This test was conducted from August to September; therefore, some variability in temperature was observed, but the temperature between systems and sections was similar on the same day. Other parameters, such as hardness (113–116 mg/L), total organic carbon (TOC) (0.5–1.8 mg/L), residual chlorine (not detected, <0.02 mg/L), and ammonia (not detected, <0.01 mg/L) in the dechlorinated tap water used in this artificial stream mesocosm were monitored quarterly (Korea Testing and Research Institute, Changwon-si, Korea), to determine whether the water quality was acceptable for aquatic organisms [44]. The benzyl chloride concentrations of surface water in each section of the system were measured after 1, 5 and 22.5 h of chemical exposure. Additionally, the concentration was measured at 48 h after exposure to check for residues of benzyl chloride. All samples were measured immediately after pre-treatment using high-performance liquid chromatography (column: Hydrosphere C18, 150 × 4.6 mm, 5 μm, Mobile phase: 80/20 (ACN/water)).

### 2.5. Animal Welfare and Institutional Animal Care and Use Committee

Fish were handled according to the standard operating procedures of the Gyeongnam Department of Environmental Toxicology and Chemistry, Korea Institute of Toxicology (KIT), and the study plan. All procedures in this study complied with the Animal Welfare Act and the Guide for the Care and Use of Laboratory Animals. This study was reviewed and assessed by the Institutional Animal Care and Use Committee (IACUC) of the Gyeongnam Department of Environmental Toxicology and Chemistry, KIT. The study was approved by the IACUC (approval number 2009-0007).

### 2.6. Statistical Analyses

The experimental analysis results of the data collected from the treatments and the control were evaluated using analysis of variance. Normality and homogeneity of variance test were determined using the Shapiro–Wilk test and Levene’s test. If these were satisfied, a one-way analysis of variance and post-hoc analysis (Dunnett’s test) were performed. Based on the statistical analysis, NOEC (No Observed Effected Concentration) was determined using the CETIS™ v. 1.8.7.16 (Tidepool Scientific Software, McKinleyville, CA, USA). The benzyl chloride concentration for the NOEC calculation was based on the mean measured concentration in each section.

### 2.7. Estimation of Hazard Concentration

HC (hazardous concentration) values were generated from the determination of the species sensitivity distribution (SSD). SSD analysis was performed using the Chemical Aquatic Fate and Effects (CAFE) database (version 1.2, National Oceanic and Atmospheric Administration, Office of Response and Restoration, Emergency Response Division, Seattle, WA, USA). The CAFE database is a software program and it is possible to estimate the fate and effects of thousands of chemicals, oils, and dispersants. The CAFE database was developed to support the assessment of chemical spills in aquatic environments and it shows the estimated short exposure risk using fate and effect data obtained from multiple databases [45].

In this study, two SSD curves for benzyl chloride were generated and compared using the CAFE program to estimate the ecological risks after a benzyl chloride spill. For the SSD created using its own database, there were few acute data points for benzyl chloride (data for only two species were available in the database), hence, it was not possible to create an SSD curve containing toxicity data. Therefore, an SSD curve was created based on interspecies correlation estimate (ICE) modeling based on existing data for *D. magna*. This program also allows users to add data; therefore, another SSD was generated using the NOEC values obtained from this mesocosm study for comparison with the integral CAFE toxicity data.

## 3. Results and Discussion

### 3.1. Benzyl Chloride Concentration in Water

Based on the physicochemical properties of benzyl chloride, the fraction of distribution was estimated using Level III fugacity modelling by EpiWin (US EPA) and the results showed that benzyl chloride remains predominantly in the water after its release (Table 3). Benzyl chloride was released into the water directly in this study, therefore it was expected that most of the benzyl chloride was distributed in the water according to this estimation result. However, hydrolysis could be the primary removal mechanism because the hydrolysis half-life (DT50) of benzyl chloride is 9.5 h at pH 7 and 25 °C [46].

In addition, an estimated Koc value of 100 determined from a logKow of 2.3 [47] indicated that benzyl chloride would not be adsorbed into the soil and sediment. Volatilization from the water surface was expected based on an estimated Henry’s Law constant of 4.12 × 10^−4^ atm·m^3^/mol, derived from a high vapor pressure of 163.9 Pa [48]. An estimated bioconcentration factor of 18 L/kg (calculated using log Kow 2.3) and biomagnification factor of 1 kg/kg (calculated using the default value of log Kow that is <4.5) indicated that the potential bioaccumulation of aquatic organisms was low [46].

Benzyl chloride was directly exposed to the water in this study, and most of the dissolved benzyl chloride and the degraded product remained in the water phase. The mean measured concentrations until the termination of chemical exposure (22.5 h) in each section are summarized in Table 4. Constant concentrations of water were maintained during exposure; however, most of the benzyl chloride could be evaporated into the air. After exposure, benzyl chloride rapidly diluted in the water, and was not detected in any section after 48 h. The mean measured concentration of each section was used to calculate the NOEC values for each species. However, to simplify, the concentrations in figures, tables and descriptions were expressed as nominal concentrations, as the real concentration varied in time and between sections. 

### 3.2. Effect of Test Organisms

- Periphyton algae

**Figure 1:**
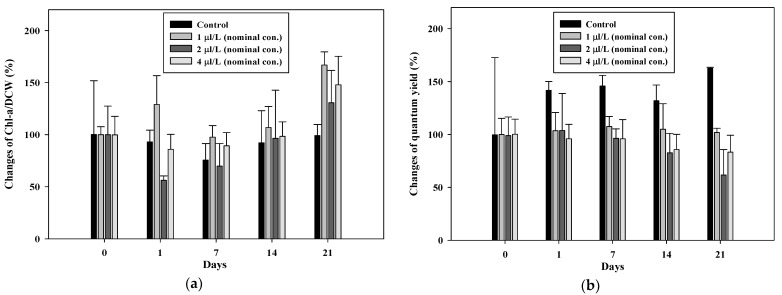
Changes in terms of (**a**) chlorophyll-a/dry cell weight and (**b**) quantum yield.

In this study, the chlorophyll concentrations in 2 µL/L and 4 µL/L treatments (mean measured concentration of the tile section: 0.41 and 0.76 µL/L, respectively) were lower until day seven, after which they gradually increased, with values ranging above those of the control (Figure 1a). However, interestingly, the quantum yield increased in the control, whereas it decreased in the 2 and 4 µL/L treatments from day one (Figure 1b). Chlorophyll is a useful indicator of photosynthesis; however, Zou and Zhang [49] recommended quantum yield measurements for samples under natural conditions because of variation problems. The results indicated that benzyl chloride was assumed to affect the photosynthetic efficiency, although the chlorophyll concentration was not diminished.

- Crustacea

**Figure 2:**
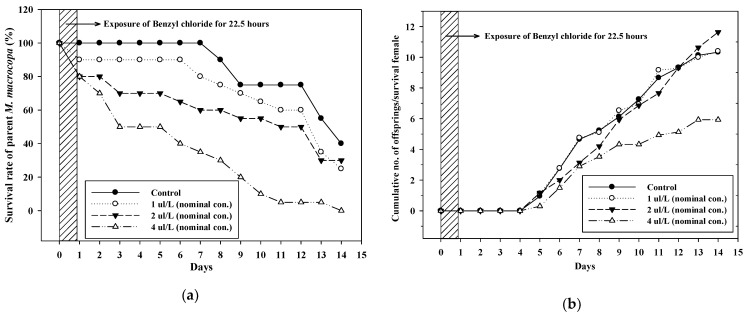
(**a**) Survival rate (%) and (**b**) cumulative number of *M. macrocopa* offspring.

The total number of offspring and the cumulative number of offspring per surviving female of *M. macrocopa* for 14 d are shown in Figure 2 and Table 5. The overall survival rate was determined on day 12 because the number of surviving individuals in all treatments groups (including the control) also decreased after 12 d. The survival rate of *M. macrocopa* decreased in all three mesocosm systems, and mortality in the 4 µL/L treatment group (mean measured concentration of gravel section: 0.98 µL/L) increased remarkably from day two, after which all organisms were dead. NOEC values were 0.28 µL/L for 1–7 d and 0.52 µL/L for 8–12 d based on the mean measured concentration. According to the ECOTOX database [50], the lowest acute toxicity value (24 h-EC50) of *D. magna* was 1.3 mg/L. Based on this value and the findings of this study, *M. macrocopa* was found to be more sensitive to benzyl chloride than the other standard species.

Reproduction initiated on day five in all mesocosm systems, and there was no difference in the initial reproductive age between the treatment group. The total number of offspring decreased in the 2 µL/L treatment group (mean measured concentration: 0.58 µL/L); however, the cumulative number of offspring based on surviving females decreased in the 4 µL/L treatment group (mean measured concentration: 0.98 µL/L). The decrease in the number of offspring in the former mesocosm could have been caused by mortality of parents because the daily average reproduction rate per surviving female (Table 5) showed that there was no significant difference in all between the mesocosm systems. Further, the NOEC was 0.52 µL/L for survival rate and 0.28 µL/L for reproduction (0.98 µL/L for reproduction rate/surviving female/day).

- Fish

**Figure 3:**
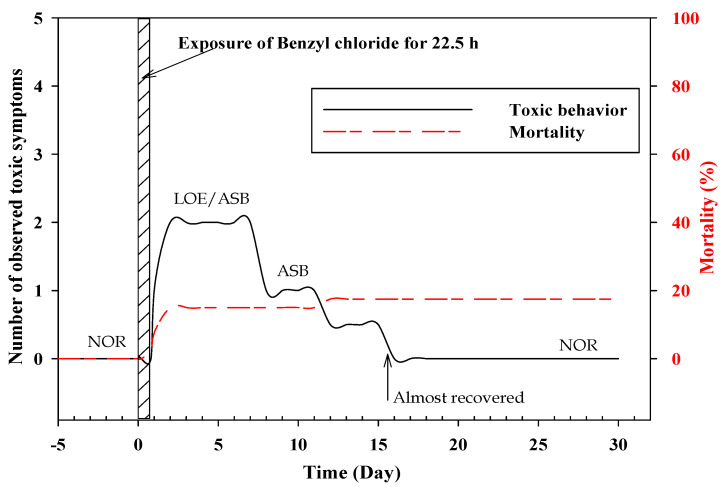
Survival rate and toxic behaviors of *Z. platypus* in the 4 µL/L benzyl chloride. (LOE: loss of equilibrium, ASB: abnormal swimming behavior, NOR: normal).

The mortality of *A. chinensis* did not occur owing to toxicity, and no specific toxicity symptoms were observed in any of the mesocosm systems within 30 d. However, *Z. platypus* mortality was 17.5% in the 4 µL/L treatment group, and toxicity symptoms such as loss of equilibrium and abnormal swimming behavior were observed for approximately 13 d, even when the organisms were no longer exposed to benzyl chloride. After 11 d, the organisms showed increased activity, and they appeared to recover after 15 d (Figure 3).

The acute toxicity data of the fish species (Table 6) showed that these species were more sensitive than other international standard species, such as *Pimephales promelas* and *Danio rerio* (toxicity range of 3–16 mg/L) according to the ECOTOX database [44]. In this study, *Z. platypus* showed delayed toxic symptoms with a recovery pattern after the termination of the chemical exposure. *A. chinensis* did not show any toxic behaviors, thus, *Z. platypus* can potentially be used as a sentinel species to study the effects of chemicals on fish. Further, no significant differences were observed in the condition factors of the two fish species on day 30.

- Chironomidae and freshwater red worm

**Figure 4:**
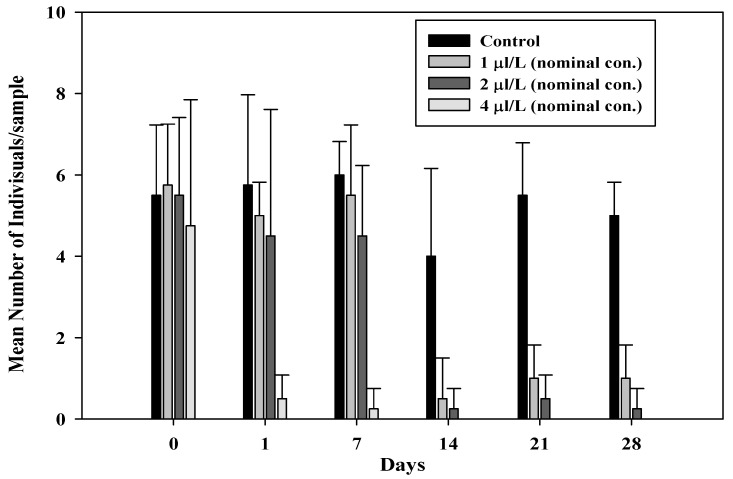
Mean number of *G. tokunagai* individuals/sample after benzyl chloride exposure.

**Figure 5:**
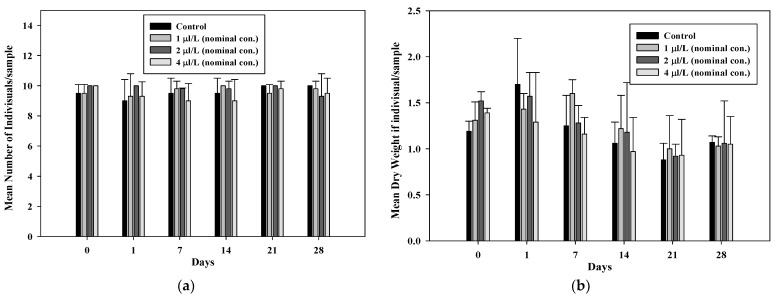
(**a**) Mean number and (**b**) dry weight of *L. hoffmeisteri* individuals/sample after benzyl chloride exposure.

The average numbers of *G. tokunagai* and *L. hoffmeisteri* individuals per sample are shown in Figure 4 and Figure 5. After benzyl chloride exposure for 22.5 h, a few *G. tokunagai* individuals were observed in the 4 µL/L treatment (mean measured concentration: 0.74 µL/L) and none were observed after seven days. In the 1 µL/L and 2 µL/L treatments (mean measured concentration: 0.25 µL/L and 0.39 µL/L, respectively), the number of individuals decreased drastically from day seven; however, 4–5 individuals continued to be observed in the control until day 28. It was assumed that almost all individuals introduced into the mesocosm systems, except the control, were dead before emergence because adults were not observed until day 28, and the developmental time (from larva to adult) of *G. tokunagai* is approximately 26–38 d [32].

Moreover, on average, 9–10 individuals of *L. hoffmeisteri* were observed per subsample in all mesocosm systems, with no toxic effects (Figure 5a). The weight of *L. hoffmeisteri* decreased in all systems after seven days, with no significant difference between the control and the treatments (Figure 5b).

### 3.3. Estimation of Hazard Concentration

**Figure 6:**
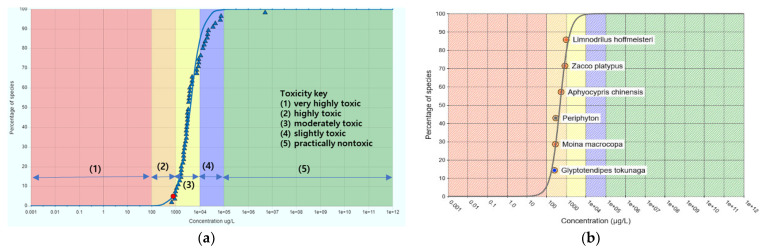
Species sensitivity distribution (SSD) curves with toxicity data. (**a**) SSD (ICE modeling data) and (**b**) SSD (mesocosm study data).

NOECs for each species are provided in Table 7 and were used for SSD analysis. Two SSD curves created using the CAFE program, using its own database and our mesocosm study data, are shown in Figure 6.

The SSD constructed using ICE modeling data showed that the toxicity was primarily located in the moderately toxic to slightly toxic range; however, the SSD acquired through our mesocosm data showed a highly toxic range. Using these SSD curves, the estimated 5th percentile HC5 values were calculated as 798 µg/L for the ICE modeling data and 94 µg/L for our study data.

## 4. Conclusions

To our knowledge, this is the first artificial stream mesocosm study simulating a benzyl chloride spill that demonstrated toxic effects on indigenous Korean aquatic species. The short-term exposure resulted in NOEC values ranging from 0.25 to 0.98 µL/L. The responses of the test species to benzyl chloride showed that *G. tokunaga* and *M. macrocopa* had NOECs ranging from 0.25 to 0.28 µL/L and were more sensitive than the other species, and that *M. macrocopa*, *G. tokunagai*, and *Z. platypus* showed delayed toxic effects. Although there was no evidence regarding the mechanisms causing delayed effects, the toxicity of benzyl chloride to aquatic organisms was evident.

According to the SSD analysis, all the Korean indigenous species selected for this study showed high sensitivity and toxicity to benzyl chloride compared to the international standard species. The findings indicated that some species can be suggested as sentinel species or environmental indicators because of their high sensitivity to benzyl chloride. The individual toxic effects of chemical spills were investigated successfully in this study, and future studies should include measurements of toxicity at the population level to simulate natural conditions more efficiently. In conclusion, this artificial stream mesocosm study can assist in risk assessments for aquatic ecosystems after chemical spills and could provide substantial evidence to establish different criteria for ecological risk assessment because some species were particularly sensitive to benzyl chloride.

## Figures and Tables

**Table 1:** Details of each section of the artificial stream mesocosm system.

Section	Size (cm) (Width × Length × Depth)	Substrate
Head tank	50 × 100 × 100	Water (80 cm depth)
Upper Riffle section	30 × 200 × 10 (with slope approximately 4.3%)	Stone/tile (0.5 cm depth for tile, approximately 10 cm depth for stone)
Lower Run section	30 × 200 × 10	Gravel (2–5 cm depth)
Pool section	30 × 100 × 40	Water (30 cm depth)
Lower section	40 × 200 × 20	Sand/sediment (10 cm depth)
Tail tank	40 × 100 × 50	Water (30 cm depth)

**Table 2:** Summary of test species.

Section	Test Species	Age	Acclimation Period	Number of Individuals	Endpoint	Study Parameter
Upper Riffle section	Periphyton algae (species not classified)	30 days growth on tiles	30 d	-	Growth	Chlorophyll-a, dry cell weight, quantum yield
dower Run section	*Moina macrocopa*	Neonates (<24 h)	-	20 individuals (5/subreplicate, 4 subrep.)	Survival, fecundity	Mortality of parents, Number of living offsprings
Pool section	*Zacco platypus*	Approximately 2–3 months (4–5 cm in length)	14 d	50 individuals	Survival, behavior, growth	Mortality, behavior observation, length and weight
Lower section	*Glyptotendipes tokunagai*, *Limnodrilus hoffmeisteri*	2nd instar larvae (*G. tokunagai*), adults (*L. hoffmeisteri*)	1 d (*G. tokunagai*) 7 d (*L. hoffmeisteri*)	240 individuals	Survival	Number of survived organisms
Tail tank	*Aphyocypris chinensis*	Approximately 2–3 months	14 d	50 individuals	Survival, behavior, growth	Mortality, behavior observation, length and weight

**Table 3:** Estimated distribution of benzyl chloride after its release in water.

Released to	Fraction of Distribution to Each Medium (% of Total)			
	Air	Water	Soil	Sediment
Water (100%)	7.9	90	0.16	1.6

**Table 4:** Mean measured concentration of benzyl chloride in water in each section.

Section	Mean Measured Concentration (±SD) (μL/L)		
	1 µL/L	2 µL/L	4 µL/L
Head Tank	0.29 ± 0.17	0.58 ± 0.32	1.01 ± 0.08
Upper Riffle section (Stone/tile)	0.29 ± 0.17	0.41 ± 0.41	0.76 ± 0.33
Lower Run section (Gravel)	0.28 ± 0.15	0.52 ± 0.33	0.98 ± 0.04
Pool section	0.27 ± 0.14	0.45 ± 0.29	0.85 ± 0.09
Lower section (Sand/sediment)	0.25 ± 0.13	0.39 ± 0.26	0.74 ± 0.13
Tail tank	0.19 ± 0.13	0.27 ± 0.22	0.54 ± 0.23

**Table 5:** *M. macrocopa* survival rate after exposure to different concentrations of benzyl chloride in stream mesocosms.

Nominal Concentration (µL/L)	^a^ Survival Rate (±SD) (%)	^a^ Total No. of Offspring	Average Offspring/Surviving Females/Day (±SD)
Control	75.0 ± 25.0	159	1.03 ± 0.51
1	60.0 ± 22.4	142	1.04 ± 0.71
2	50.0 ± 25.0	104 *	1.16 ± 0.31
4	5.0 ± 11.2 *	38 *	0.59 ± 0.45

* Significant differences (*p* < 0.05). ^a^ based on the data at day 12.

**Table 6:** Survival rates and condition factors in *Z. platypus* and *A. chinensis,* following benzyl chloride exposure.

Nominal Concentration (µL/L)	Survival Rate (%)		Condition Factor (K) (±SD)	
	*A. chinensis*	*Z. platypus*	*A. chinensis*	*Z. platypus*
Control	100	97.5	1.09 ± 0.15	1.05 ± 0.26
1	100	100	1.10 ± 0.16	0.92 ± 0.18
2	97.5	100	1.09 ± 0.23	0.92 ± 0.19
4	100	82.5	1.05 ± 0.19	0.95 ± 0.21

**Table 7:** No observed effect concentration (NOECs) for each species.

NOEC (μL/L)					
*G. tokunaga*	*M. macrocopa*	Periphyton Algae	*A. chinensis*	*Z. platypus*	*L. hoffmeisteri*
0.25	0.28	0.29	0.54	0.85	0.98

## Data Availability

Not applicable.

## Supplementary Materials

The following are available online at https://www.mdpi.com/article/10.3390/toxics9120347/s1, Table S1: Water temperature (°C).
