# Supplementary material for: Ecological Effects of Benzyl Chloride on Different Korean Aquatic Indigenous Species Using an Artificial Stream Mesocosm Simulating a Chemical Spill
Source: Toxics. 2021 Dec 9;9(12):347. doi: 10.3390/toxics9120347 (PMC8703321; doi:10.3390/toxics9120347)
Supplement: Supplementary file 1 [file toxics-09-00347-s001.zip › toxics-1430833-supplementary.pdf]

toxics MDPI

Supplementary Materials: Ecological Effects of Benzyl Chloride on Different Korean Aquatic Indigenous Species Using an Artificial Stream Mesocosm Simulating a Chemical Spill

Sooyeon Kim, Seong-Hwan Park, Dae-Wook Kim, Won Noh, Sang-Jun Lee, Hee-Jin Jeong, Jong-Bin Park, Yeong-Ji Gwak, Jin-Woo Park and Dong-Hyuk Yeom

Table S1. Water temperature (°C).

		Sample 1					Sample 2					Sample 3					Sample 4				
**Date Time**	**Day**	**1**	**2**	**3**	**4**	**5**	**1**	**2**	**3**	**4**	**5**	**1**	**2**	**3**	**4**	**5**	**1**	**2**	**3**	**4**	**5**
11/08 10:00	0	23.2	23.3	23.4	23.5	23.5	23.2	23.3	23.4	23.6	23.5	23.3	23.4	23.5	23.6	23.5	23.3	23.4	23.4	23.5	23.5
12/08 9:20	1	23.8	23.9	24.0	24.1	24.2	24.0	23.9	24.1	24.1	24.2	23.9	23.9	24.1	24.2	24.3	23.9	24.0	24.1	24.2	24.3
18/08 15:00	7	23.6	24.3	25.4	26.1	27.0	23.8	24.8	25.7	26.3	27.2	23.7	24.4	25.5	26.0	26.9	23.8	24.4	25.8	26.3	27.4
25/08 15:00	14	24.4	24.7	25.6	26.3	27.1	24.7	25.0	25.9	26.7	27.4	24.4	25.3	25.9	26.4	27.3	24.6	25.7	26.4	26.9	27.9
01/09 16:30	21	24.0	24.1	24.2	24.3	24.8	23.9	24.0	24.2	24.4	24.8	23.3	23.8	24.3	24.4	24.9	23.1	23.5	24.3	24.5	25.0
08/09 17:00	28	23.5	23.1	23.9	24.1	24.6	23.2	23.4	23.9	24.1	24.5	23.8	23.9	23.9	24.2	24.6	23.7	23.8	23.8	24.1	24.6
10/09 10:00	30	22.8	23.4	23.0	23.0	23.2	22.9	23.6	23.0	23.1	23.3	23.3	23.4	23.5	23.5	23.4	23.1	23.3	23.5	23.5	23.5

1: Upper riffle section. 2: Lower Run section 3: Pool section 4: Lower section 5: Tail tank
